# Metagenome-informed metaproteomics methodology: a new breakthrough in revealing the mechanisms of host-microbiota-diet interactions in inflammatory bowel disease

**DOI:** 10.3389/fcell.2025.1634974

**Published:** 2025-07-21

**Authors:** Suqin Zhang, Qi Xu

**Affiliations:** ^1^Department of Neurosurgery, Sinopharm Tongmei General Hospital, Datong, China; ^2^Department of Gastroenterology, Second Affiliated Hospital of Dalian Medical University, Dalian, China

**Keywords:** metagenome-informed metaproteomics, inflammatory bowel disease, microbiota, dietary, metaproteomics

## Introduction

Inflammatory bowel disease (IBD), a chronic recurrent intestinal inflammatory disease, has become a major digestive system disease that seriously endangers human health (Kaplan and Ng, 2017). Studies have shown that intestine microbiota disorders and dietary factors have synergistic promoting effects on the course of IBD (Godala et al., 2022). The human intestinal system is essentially a dynamic metabolic network composed of host-microbiota-dietary elements, in which dietary factors can participate in the disease process through multiple pathways, such as regulating the function of the mucosal barrier, reshaping structure of the microbiota, and affecting the expression of metabolic proteins (Sugihara and Kamada, 2021). In clinical practice, the therapeutic approach for IBD management typically prioritizes enteral nutrition supplementation combined with microbial regulators to alleviate patient symptoms (Levine et al., 2020; Roy and Dhaneshwar, 2023). While these empirical treatment protocols demonstrate clinical efficacy in symptom control, their mechanistic limitations hinder progress in understanding IBD pathogenesis. This knowledge gap primarily stems from insufficient characterization of the dynamic triad interactions between host physiology, gut microbiota composition, and dietary influences. In that background, Valde’s-Mas et al. innovatively developed a metagenome-informed metaproteomics (MIM) technology, which for the first time achieved synchronous dynamic monitoring of host regulatory proteins, microbial functional proteins, and dietary residual proteins (Valdés-Mas et al., 2025). This technology integrates multi-dimensional omics data, constructs a dynamic network model of the interaction between the three. It reveals the spatiotemporal variation rules of key biomarkers in the course of IBD, but also provides a new research paradigm for in-depth analysis of the molecular of this disease.

## Result

Traditional microbiological research heavily relied on DNA sequencing technology, making it difficult to dissect microbial activity characteristics dominated by protein functions. MIM technology proposed in this study, by constructing an experimental specific protein reference database, has realized the simultaneous quantitative analysis of host immune proteins, microbial proteins, and dietary residues proteins in the intestine. To minimize misidentification of bacterial proteins caused by protein homology, the research team designed a multi-level filtering strategy and make it possibile to draw a specific protein map for the intestine. To verify the reliability of the strategy, the specific map of intestine were drawn in antibiotic users and healthy individuals. The consistent bacterial density gradient in healthy intestine, the progressive increase in bacterial protein quantity and species diversity along the distal gastrointestinal tract, were also identified which is consistent with metagenomic analysis. Notably, the differences of MIM-based niche-specific protein commensal configurations were also identified. For example, the significant enrichment of carbohydrate utilization proteins expressed by *Streptococcus* mutans in the stomach compared to feces. Furthermore, the study established a specialized dietary protein screening methodolgy to enable precisly identificate dietary proteins. In all, the MIM technology has provided a reliable methodological support for revealing the protein associated molecular mechanism of intestinal bacteria and dietary action in the development of IBD.

This study employed the MIM methodology to establish intestine-specific proteomic profiles across clinical populations and experimental models, analyzing both IBD patients and dextran sulfate sodium (DSS)-induced murine IBD systems. The clinical cohort design incorporated two distinct demographic groups: a pediatric Crohn’s disease cohort from Israeli and an adult ulcerative colitis cohort from German. Finally, several dysbiosis patterns in IBD were identified. The compositional dysbiosis pattern, characterized by quantitative imbalances in symbiotic microbiota, comprises two subtypes: one can be detectable by both MIM and metagenomic methods (e.g., *S. copri*, *L. rogosae*, and *Akkerman sia muciniphila*), and another pattern specially identified by MIM but not metagenomic analysis (e.g., *Faecalibacterium prausnitzii*, *Fusicatenibacter saccharivorans*, and *Alistipes putre dinis*). Functional dysbiosis pattern, defined by altered protein expression profiles without corresponding metagenomic abundance changes, was observed in: *Bifidobacterium adolescentis* and *Bacteroides caccae*, which maintained stable genomic representation but demonstrated significant proteomic perturbations in MIM analyses. Fecal microbiota transplantation (FMT) from healthy controls or gastroesophageal reflux-associated ulcerative colitis (GER-UC) patients into germ-free (GF) mice was performed to investigate microbial regulation of host intestinal protein secretion in colitis pathogenesis. Recipient mice colonized with UC microbiota exhibited both constitutive and functional dysbiosis patterns. However, no clinical manifestations of colitis or systemic inflammation were detected post-colonization, suggesting that microbiota alterations alone are insufficient to initiate overt autoinflammatory responses in the absence of host genetic predisposition. Complementary fecal dietary proteomic analyses revealed elevated total dietary protein levels in IBD patients with upper gastrointestinal tract involvement compared to healthy controls or those with ileocolonic-restricted disease (endoscopically confirmed). This observation implies impaired small intestinal absorption may drive luminal protein accumulation. Collectively, these findings establish novel mechanistic links between gut microbiota dynamics and IBD progression.

This study tend to explore protein biomarkers for inflammatory bowel disease (IBD) through a novel diagnostic framework combining host-microbial proteomic profiling. Leveraging the MIM methodology, they identified the top 50 discriminatory protein features from host and microbial proteomes in the HMP-MGH and HMP-CinHMP cohorts, generating various single/double-marker panels. Subsequent validation across three independent cohorts employed repeated five-fold cross-validated random forest modeling to evaluate diagnostic performance against the conventional biomarker calprotectin. Their analysis revealed that multiple bacterial-host protein pairs demonstrated superior diagnostic accuracy compared to calprotectin. Notably, a biomarker panel containing lactoferrin (LTF) and the bacterial phosphopyruvate hydratase (ppdK, K01006) - a core glycolytic enzyme - achieved enhanced discrimination of both ulcerative colitis (UC) and Crohn’s disease (CD). These findings establish a proteomic evaluation system with translational potential for IBD prediction and progression monitoring.

## Significance of the study

This study demonstrates three groundbreaking advances through the innovative implementation of MIM technology. First, the MIM methodology achieves superior sensitivity in detecting functional microbial proteins, enabling precise tracking of dietary and microbial impacts on gut proteome dynamics. By directly quantifying the dietary exposome—overcoming inherent limitations of self-reported dietary assessments such as recall bias and poor compliance, it can identify low compliance individuals in time. Furthermore, this technological breakthrough addresses the clinical gap in monitoring small intestinal dysfunction, traditionally dependent on invasive procedures like capsule endoscopy.

Second, proteomic analyses of IBD patient cohorts and model mice systematically revealed functional heterogeneity in microbial dysbiosis. While baseline proteomic profiles remained stable, which is consistent with metagenomic analysis, specific functional proteins (e.g., ABC transporters) exhibited marked expression abnormalities. MIM method detected protein-level functional defects—such as Bmp transporter inactivation in *Oscillibacter* sp. ER4 and *Subdoligranulum copri*, which were undetectable via genomic abundance measurements. These findings establish protein-specific dysfunction as a molecular criterion for defining pathogenic “driver” commensals, with clinical implications for diagnostics, microbiota transplantation therapy, and prognostic evaluation.

Third, MIM identified clinically actionable host-microbe biomarker panels that outperform the current gold standard calprotectin in IBD differential diagnosis (CD vs UC) and disease staging. Notably, certain biomarkers, such as proteins from Alistipes putredinis and *Bacteroides* vulgatus, exhibited expression changes exclusively at the proteomic level. This supports the concept of microbiome dysfunction independent of microbial population abundance, offering new perspectives for IBD mechanism research and precision diagnostics.

## Future directions

With the research paradigm for IBD pathogenesis transitioning from “microbial dysbiosis” to “intestinal protein imbalance,” MIM technology provides critical methodological support for systematically analyzing protein networks within tripartite host-microbe-diet interactions. Future investigations should prioritize these key directions: Firstly, overcoming current technological limitations by enhancing protein sequencing sensitivity, optimizing identification systems for rare microbial species, and developing personalized MIM phenotypic frameworks (Lloyd-Price et al., 2019; Gilliland et al., 2024; Schirmer et al., 2019). Furthermore, elucidating nutrient-specific metabolic pathways and characterizing dynamic correlations between disease-specific proteomic profiles across intestinal pathologies (Lloyd-Price et al., 2019). In parallel, the secreted protein and antimicrobial peptide (AMP) quantified by MIM needs further exploration in IBD (Gonzalez et al., 2017). Lastly, addressing clinical implementation barriers through cost-effective MIM adaptations to enable accessible diagnostic solutions (van Rheenen et al., 2020; Briend et al., 2022). Notably, the application of MIM technology could be extended to extra-intestinal ecosystems, such as the female reproductive tract. Comparative analysis of host-microbe functional interactions across ecological niches may unveil fundamental microbial regulatory principles underlying multifactorial diseases, offering a novel interdisciplinary research paradigm.
